# Supplementary material for: Incorporation of Lutein on Layered Double Hydroxide for Improving the Environmental Stability
Source: Molecules. 2020 Mar 9;25(5):1231. doi: 10.3390/molecules25051231 (PMC7179472; doi:10.3390/molecules25051231)
Supplement: Supplementary file 1 [file molecules-25-01231-s001.pdf]

Supplementary Informationfor Incorporation of lutein on layered double hydroxide for improving the environmental stability

Shue Li[1,2,3], Bin Mu[1,3,*], Wenkai Dong[1,2,3], Oing Liang[1,3], Shijun Shao[1,3] and Aiqin Wang[1,3,*]

[1] Key Laboratory of Clay Mineral Applied Research of Gansu Province, Center of Eco-Materials and Green Chemistry, Lanzhou Institute of Chemical Physics, Chinese Academy of Sciences, Lanzhou 730000, P. R. China

[2] Center of Materials Science and Optoelectronics Engineering, University of Chinese Academy of Sciences, Beijing 100049, P. R. China

[3] Center of Xuyi Palygorskite Applied Technology, Lanzhou Institute of Chemical Physics, Chinese Academy of Sciences, Xuyi 211700, P. R. China

* Correspondence: mubin@licp.cas.cn (B.M.); aqwang@licp.cas.cn (A.W.); Fax: +86 931 4968019 (A.W.); Tel: +86 931 4868118 (A.W.).

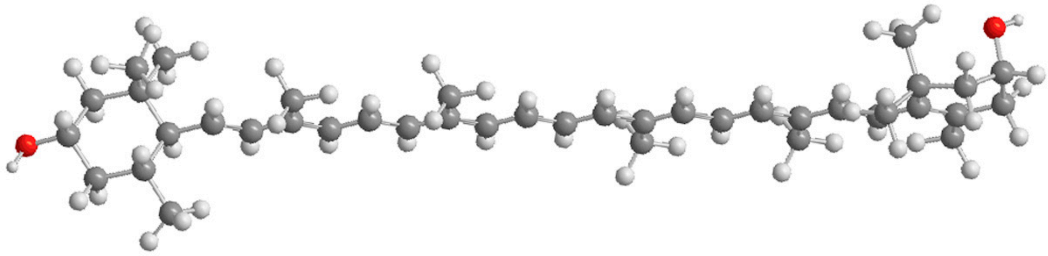

Figure S1. Structural model of lutein molecule (the gray, white and red spheres represent carbon, hydrogen and oxygen atoms, respectively).

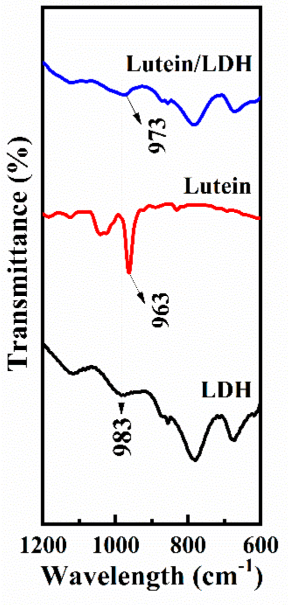

Figure S2. Partial enlarged view of FTIR spectra of lutein, LDH and lutein/LDH composites.

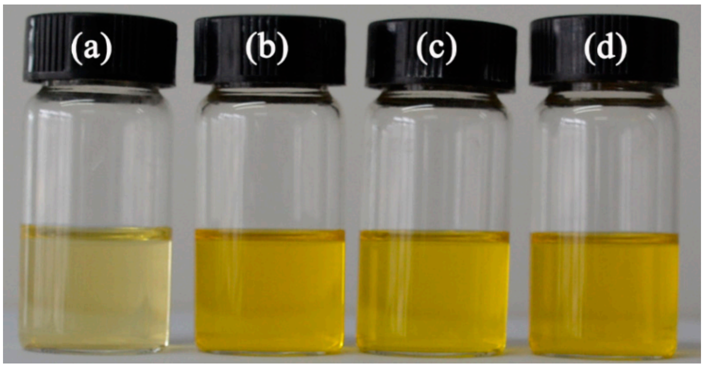

Figure S3. Digital photos of the supernatants after attacks of (**a**) 0.1 M NaOH, (**b**) acetone, (c) ethanol and (**d**) ethyl acetate for 24 h, respectively.

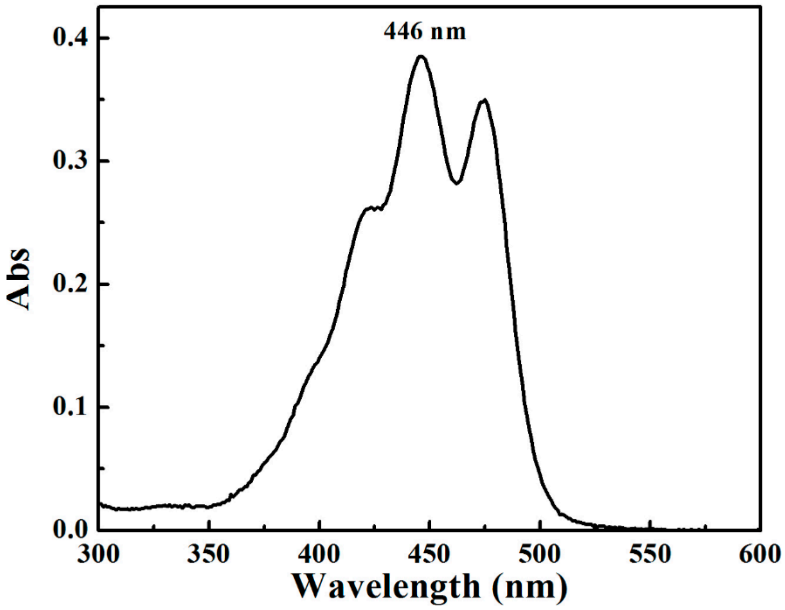

Figure S4. UV spectrum of lutein in ethanol solution.
